# Neonatal factors associated with early weaning in a municipality in Bahia, Brazil: a cross-sectional study

**DOI:** 10.1590/1980-220X-REEUSP-2024-0091en

**Published:** 2024-10-07

**Authors:** Aloysia Graça Costa Unfried, Gilvânia Patrícia do Nascimento Paixão, Chalana Duarte de Sena Fraga, Josenilde Damascena Oliveira, Jorge Lopes Cavalcante

**Affiliations:** 1 Universidade do Estado da Bahia, Salvador, BA, Brazil.; 2 Universidade do Estado da Bahia, Senhor do Bonfim, BA, Brazil.; 3 Universidade do Estado da Bahia, Jacobina, BA, Brazil.

**Keywords:** Breast Feeding, Milk, Human, Infant, Weaning, Infant Formula, Lactancia Materna, Leche Humana, Lactante, Destete, Fórmulas Infantiles

## Abstract

**Objective:**

To analyze neonatal factors associated with early weaning.

**Method:**

This is a cross-sectional study conducted between March and September 2023 with 180 women six months to two years postpartum, from a municipality in Bahia, Brazil. For bivariate analysis, Pearson’s chi-square tests were used, considering p < 0.05. The adjusted analysis included variables with p < 0.20, keeping those with p < 0.05, using stepwise multiple logistic regression, with a 95% confidence interval.

**Results:**

The provision of pacifiers/bottles (OR: 18.96; 95% CI: 7.68–46.79; p < 0.001) and supplements in the maternity ward (OR: 4.44; 95% CI: 1.76–11.17; p: 0.002) were associated with greater likelihood of early weaning.

**Conclusion:**

Habits and beliefs, such as the use of bottles and pacifiers, and the introduction of infant formulas within the maternity ward with continued supplementation after hospital discharge were the neonatal factors associated with early weaning in this context.

## RESUMEN

**Objetivo:**

Analizar los factores neonatales relacionados con el destete precoz.

**Método:**

Estudio transversal realizado entre marzo y septiembre de 2023 que incluyó a 180 mujeres con entre seis meses y dos años de posparto, en un municipio de Bahía, Brasil. Para el análisis bivariado se emplearon pruebas de chi-cuadrado de Pearson, considerando un valor de p < 0,05. El análisis ajustado incluyó variables con p < 0,20, manteniendo las con p < 0,05, mediante regresión logística múltiple por pasos, con un intervalo de confianza del 95%.

**Resultados:**

El uso de chupetes y biberones (OR: 18,96; IC 95%: 7,68–46,79; p < 0,001) y la administración de suplementos en la maternidad (OR: 4,44; IC 95%: 1,76–11,17; p: 0,002) se asociaron con una mayor probabilidad de destete precoz.

**Conclusión:**

Los hábitos y creencias, como el uso de biberones y chupetes, así como la introducción de fórmulas infantiles en la maternidad y la continuidad de la suplementación tras el alta hospitalaria, son factores neonatales asociados al destete precoz en este contexto.

## INTRODUCTION

A source of nutrients for child growth and development, human milk is composed of diverse and complex ingredients. These include macronutrients, such as carbohydrates (lactose), fats, proteins, and numerous vitamins and minerals, as well as bioactive factors, immunoglobulins, metabolites, and microorganisms with physiological roles specific to human milk^(1)^.

Studies show much evidence of its effects as early as in childhood, particularly in reducing mortality rates^(2)^ caused by respiratory tract and gastrointestinal infections^(3)^. In adulthood, there are positive effects on the development and integrity of the oral cavity, reducing the rates of dental malocclusion, in addition to other benefits, such as a lower chance of chronic comorbidities, including overweight and diabetes^(4,5)^.

The World Health Organization (WHO) and the Brazilian Ministry of Health (MH) recommend that Exclusive Breastfeeding (EB) directly from the breast or expressed, without solids, be maintained until the sixth month of life; complementary feeding should be introduced from this point onwards, with continued breastfeeding for at least two years^(6)^.

Brazil is a world reference for human breastfeeding (HB) due to public policies adopted at least 30 years ago to encourage breastfeeding^(7)^. According to the Brazilian National Food and Nutrition Study (*Estudo Nacional de Alimentação e Nutrição* – ENANI) conducted between February 2019 and March 2020, over the last 30 years there has been a significant increase in the prevalence of EB up to six months of age, increasing from 2.9% to 45.7%. Throughout this period, there was also a reduction in the incidence of diarrheal diseases and respiratory infections, while the life expectancy of Brazilian children increased^(8)^.

However, even with the improvement in the prevalence rates of EC in Brazil, the rates still do not meet current WHO recommendations of 55% of infants on EC by 2025 and 70% by 2030; EC rates are particularly low in the Northeast (39.0%) and North (40.3%) regions^(6,7,8)^.

Given the positive impacts of EB up to six months of the newborn’s life, as well as variations in prevalence rates in Brazilian regions, it is necessary to carry out more regionalized studies, with the aim of identifying and understanding the predictive factors for early weaning on an individual basis, given the numerous neonatal factors that can interfere in EB management. Despite the heterogeneity of studies and the specificities of each Brazilian region, recent studies^(9,10)^ suggest that the use of bottles and pacifiers and the early introduction of formulas are potential neonatal predictors of early weaning.

Clarity regarding such characteristics may guide the adequacy of lines of care and prenatal, postpartum, and childcare monitoring, corroborating the scientific community on the prevention of modifiable factors that may result in early interruption of breastfeeding. Therefore, the following research question was posed: what are the neonatal factors associated with early weaning? The objective was to analyze neonatal factors associated with early weaning.

## METHOD

### Study Design

This is a cross-sectional study with a quantitative approach following the Strengthening the reporting of observational studies in epidemiology (STROBE) guidelines.

### Location, Population, and Selection Criteria

The study was conducted in Senhor do Bonfim – BA, a municipality located in central-northern Bahia, Brazil, which has a territorial area of 789,361 km, 79,813 inhabitants, a demographic density of 94,413 inhabitants/km^2^ and 17.69 deaths per 1,000 live births^(11)^.

The study population consisted of 180 dyads linked to the municipality’s Family Health Strategy (*Estratégia de Saúde da Família* – ESF). Those included were women between six months and one day and two years (24 months) postpartum, with a living child, counted from the date of application of the form, over 18 years of age, and registered in the ESF of that municipality. Women who had serious mental disorders or who were unable to breastfeed due to medical recommendation were not included, as established by the ESF professionals. The sample was selected by the ESF team, which indicated eligible participants, with these criteria.

### Sample Size Calculation

The sample was calculated using a simple random sample without replacement for finite populations. The Survey Monkey calculator (https:pt.surveymonkey.com/mp/sample-size-calculator/) was used. A population size of 951 live births was considered, based on the consolidation report of the Community Health Agents (*Agentes Comunitários de Saúde* – ACS) form of the municipality of Senhor do Bonfim in 2022, 80% power and 5% alpha, leading to 140 individuals. However, considering the number of independent variables to be investigated, forty more individuals were added, totaling 180 participants. Maintaining the proportion between the urban and rural populations of the municipality, 69 and 111 women were investigated, respectively.

### Data Collection

A pilot test was conducted for data collection to test and adapt the instrument, which was based on the most recent publications on maternal and child health and guidelines from the Brazilian MH and the WHO. The test was applied to ten women meeting the eligibility criteria, readapted, given the challenges encountered during the application, and subsequently discarded.

Initially, there was a prior conversation with the nurse from each ESF unit, with a survey of women matching the target population of the study. Subsequently, contact information for the ACS team was made available. The agents scheduled the day and time to visit each woman eligible for the study through telephone contact after confirming their interest in participating in this study.

The data were collected individually between March and September 2023 by three properly trained researchers in a single meeting at the participant’s home. After the research objective was presented and participants signed two copies of the Informed Consent Form (ICF), the then unvalidated research instrument was applied. It has sixty-three closed questions (multiple choice or dichotomous) about sociodemographic and obstetric information, lifestyle habits, the infant, and professional care. The variables for this manuscript were selected based on its objective, i.e., the infant variables (neonatal data) were selected, totaling the fourteen variables described below.

The outcome variable early weaning (breastfeeding discontinuation before six months) was analyzed through the following question: Discontinued exclusive breastfeeding before six months (no, yes).

The variables related to the infant were: sex (female, male), current age (6 to 12 months, 13 to 24 months), birth weight (less than 2.5 kg, between 2.5 kg and 3 kg, between 3 and 4.85 kg), gestational age at birth between 37 and 41 weeks (no, yes), skin-to-skin contact in the first hour (no, yes), time since postpartum breastfeeding began (up to 1 hour, after 1 hour), received another type of milk in the maternity ward (no, yes), breastfeeding on demand (no, yes), baby had difficulty sucking (no, yes), received another type of milk on medical advice (no, yes), used pacifier or bottle before 6 months (no, yes), pacifier or bottle introduced in the maternity ward (no, yes), baby has required hospitalization (no, yes), had up to 6 childcare appointments (no, yes).

### Data Analysis and Processing

The data were entered into Microsoft Excel® 2010 and subsequently exported for analysis in the STATA statistical program, version 15.0 (*Stata Corp.,* USA). The independent variables with p < 0.05 in the bivariate analysis, using Pearson’s chi-square test, were selected and included in the multivariate analysis. In multivariate analyses, the independent variables whose association in the crude analysis was significant at the 20% level (p ≤ 0.20) were considered together.

As a variable selection method for the final model, stepwise for multiple logistic regression was employed. Thus, the adjusted analysis included independent variables with a value of p < 0.20 and variables that showed a statistically significant association with the early weaning outcome at a level of 5% (p < 0.05) were maintained in the final model.

Crude estimates of odds ratios (OR) referring to the association between the dependent variable (early weaning) and independent variables (neonatal factors) were thus obtained, as well as the odds ratios adjusted in the final model and their respective 95% confidence intervals (95% CI).

### Ethical Aspects

This study is linked to the Research and Extension Project Breastfeeding Support Group (*Grupo de Apoio ao Aleitamento Materno* – GAAM), developed at *Universidade do Estado da Bahia*, Campus VII, and was approved by the Research Ethics Committee of *Universidade do Estado da Bahia* in 2022 under opinion number 5,437,925 and CAAE 84122117.8.0000.0057. The Informed Consent Form (ICF) was provided to all participants and the ethical principles of research of the National Health Council’s Resolution No. 466/12 were respected.

## RESULTS

This study included 180 women. Most were aged 16 to 29 years, had a high school level, were married or in a stable union, declared themselves mixed race or black, carried out household activities, and reported receiving assistance from social programs. They lived in an urban area with their husbands and children, required no transportation to go to the basic health unit, and had been presented with information about breastfeeding through prenatal consultations. Consumption of alcohol or cigarettes during pregnancy/postpartum was reported by 13.89% of those interviewed. The prevalence of early weaning in the studied sample was 48.33% (n = 87).

Table 1 presents the crude analysis of the association between neonatal characteristics and early weaning. The following factors were positively associated with the outcome: used a pacifier and/or bottle (OR: 16.36; 95% CI: 7.06–37.88; p < 0.001); introduced pacifiers and/or bottles in the maternity ward (OR: 3.58; 95% CI: 0.13–0.57; p: 0.001); received other milk on medical advice (OR: 14.72; 95% CI: 1.8–115.8; p: 0.001); received another type of milk in the maternity ward (OR: 3.28; 95% CI: 1.60–6.71; p: 0.001), time since breastfeeding began postpartum (OR: 0.41; 95% CI: 1.29–4.46; p: 0.005); breastfeeding carried out on demand (OR: 0.14; 95% CI: 0.01–1.24; p: 0.044).

**Table 1:** Crude analysis of infant characteristics according to early weaning* – Senhor do Bonfim, BA, Braszil, 2023 (n = 180).

Neonatal characteristics	Early weaning				P	Crude OR (95% CI)
	No		Yes			
	n	%	N	%	
**Infant sex**					0.762		
Female	47	52.81	42	47.19		1	
Male	46	50.55	45	49.45		1.09(0.60–1.96)	
**Current child age**					0.821		
Six months to one year	38	50.67	37	49.33		1	
Thirteen to twenty-four months	55	52.38	50	47.62		0.93(0.51–1.68)	
**Weight at birth**					0.406		
Under 2,500 g	09	50.00	09	50.00		1	
Between 2,500 g and 3,000 g	23	44.23	29	55.77		1.26(0.43–3.69)	
Between 3,000 g e 4,850 g	61	55.45	49	44.55			
**Gestational age at birth was 37 to 41 weeks**					0.492		
No	06	42.86	08	57.14		1	
Yes	87	52.41	79	47.59		0.68(0.22–2.04)	
**Skin-to-skin contact within the first hour**					0.077***		
No	21	41.18	30	58.82		1	
Yes	72	55.81	57	44.19		0.55(0.28–1.06)	
**When breastfeeding was started postpartum**					0.005**		
Up to 1 hour postpartum	67	59.82	45	40.18		1	
After 1 hour postpartum	26	38.24	42	61.76		2.40(1.29–4.46)	
**Received a different kind of milk at the maternity ward**					0.001**		
No	79	58.96	55	41.04		1	
Yes	14	30.43	32	69.57		3.28(1.60–6.71)	
**Breastfed on demand**					0.044***		
No	01	14.29	06	85.71		1	
Yes	92	53.18	81	46.82		0.14(0.01–1.24)	
**Baby had sucking problems**					0.218		
No	82	53.59	71	46.41		1	
Yes	11	40.74	16	59.26		1.67(0.73–3.85)	
**Received another kind of milk on medical advice**					0.001**		
No	92	55.09	75	44.91		1	
Yes	01	07.69	12	92.31		14.72(1.8–115.8)	
**Baby has been hospitalized**					0.321		
No	79	49.69	80	50.31		1	
Yes	08	38.10	13	61.90		0.62(0.24–1.58)	
**Used pacifier or bottle**					<0.001**		
No	58	87.88	08	12.12		1	
Yes	35	30.70	79	69.30		16.36(7.06–37.88)	
**Pacifier and/or bottle introduced in the maternity ward**					0.001**		
No	80	59.26	55	40.74		1	
Yes	13	28.89	32	71.11		3.58(0.72–7.43)	
**Has been to up to six childcare consultations**					0.496		
No	52	49.52	53	50.48		1	
Yes	41	54.67	34	45.33		0.81(0.44–1.47)	

Note: *Exclusive breastfeeding discontinued before six months of age

**p < 0.05.

***p < 0.20.

In Table 2, the adjusted multiple logistic regression analysis of neonatal aspects associated with the outcome of early weaning is presented. It was found that infants who used pacifiers and/or bottles and those who received another type of milk while still in the maternity ward were 18.96 (95% CI: 7.68–46.79; p < 0.001) and 4.44 (95% CI: 1.76–11.17; p: 0.022) times more likely to present early weaning. This final logistic regression model was able to explain in 28% (chi^2^ = 0.2868) the association of the independent variables (Used a pacifier and/or bottle/Received another type of milk in the maternity ward) with the dependent variable (early weaning).

**Table 2:** Adjusted logistic regression analysis of neonatal factors
associated with the outcome of early weaning* – Senhor do Bonfim, BA, Brazil, 2023 (n = 180).

Variables	N (%)	Adjusted OR (95% CI)	P
**Used pacifier and/or bottle**			<0.001
No	66(36.67)	1	
Yes	114(63.33)	18.96(7.68–46.79)	
**Received a different type of milk in the maternity ward**			0.002
No	134(74.44)	1	
Yes	46(25.56)	4.44(1.76–11.17)	

Note: *Exclusive breastfeeding discontinued before six months of age.

## DISCUSSION

The objective of this study was to analyze the neonatal factors associated with early weaning in a sample of 180 women from the interior of the State of Bahia, Northeastern Brazil. Among the neonatal factors analyzed in this study, the use of pacifiers and/or bottles and having received another type of milk while still in the maternity ward were positively associated with early weaning.

The percentage of infants who weaned early was 48.33%, lower than generally estimated in the Brazilian context, which correspond to 54.3%^(8)^. This important finding revealed that more than half of the infants were exclusively breastfed until 6 months of age (51.67%). This shows that, in the region evaluated in this research, the exclusive breastfeeding rate was higher than that found in Brazil between 1986 and 2020^(8)^.

Among the variables that were positively associated with the study outcome, the use of pacifiers and/or bottles was most strongly associated with early weaning. Thus, women who reported providing them to infants had 18.96 times the chance of weaning their child early when compared with those who did not offer artificial teats. The prevalence of the use of artificial teats among infants under 24 months is 52.1% in Brazil and 55.8% in the Northeast region^(8)^. In our study, the use of pacifiers and/or bottles had a prevalence of 63.34%.

Exclusive breastfeeding may be discontinued due to socioeconomic and health factors, in addition to biological factors^(10)^. The literature has pointed out that the lack of encouragement from health institutions, lack of family support for nursing mothers, and the provision of artificial teats (pacifiers and/or bottles) are among the main causes of early weaning^(12,13)^.

The reasons for introducing pacifiers need to be defined. Over the generations, pacifiers were culturally linked to quieting the baby’s crying. However, in addition to a confusion of beaks, using pacifiers to calm down infants can directly interfere with the production and quantity of human milk, as it is directly related with a reduction in breastfeeding; in other words, the greater the frequency of breastfeeding, the higher the milk production^(14,15)^.

The Brazilian scenario does not differ from the international one in relation to the negative influence of pacifier use. A study conducted in Rome with 542 women identified that infants who used pacifiers in the first two weeks of life had a 2.39 times greater risk of discontinuing breastfeeding when compared to those who did not^(16)^.

In addition to the complication of early weaning, the use of artificial teats promotes changes in the breathing pattern, favoring mouth breathing, which leads to impaired speech and malocclusion, such as open bite or crossbite. Among other negative aspects for the infant’s health are episodes of acute otitis media, changes in the palate, jaw, and immunological impairment, as they are considered potential reservoirs of contamination, causing fever, diarrhea, mouth ulcers, and candidiasis^(15,17)^.

Such results provide evidence for the need to discourage the use of pacifiers within professional practice, aiming to maintain EB for the recommended time^(12)^. Furthermore, use of artificial teats is not recommended, as it represents a means of contamination, interferes with breastfeeding on demand, reduces breast sucking time, and affects the natural development of swallowing, in addition to delaying the time it takes to establish lactation^(6,18)^.

Having received another type of milk in the maternity ward was also strongly associated with the outcome and predictive of early weaning in the studied population. These infants had a 4.44 times greater chance of being weaned early when compared to those who were fed human milk during this period. In this study, a prevalence of 25.56% of infants who received supplements in the first hours postpartum was found.

Supplement is characterized by the offer of pasteurized human milk or infant formula, in the absence of the former, to newborns (NB) under different conditions^(19)^. Despite efforts to maintain exclusive breastfeeding, the offer of complementary feeding within the first hours of a low-risk NB, with a predominance of infant formulas, is still a frequent practice without justifiable reasons, according to the Baby Friendly Hospital Initiative (BFHI)^(20)^.

National and international institutions routinely use supplements postpartum^(15)^, which is evidenced in several studies, namely: public maternity hospitals in the city of Natal, Rio Grande do Norte, showed a frequency of 57.6% in the use of supplements in the first hour of life^(19)^; a maternity hospital in Rio de Janeiro, with a prevalence of 12% of NB who received dairy supplements during hospitalization^(21)^, in addition to maternity hospitals in Iran and five hospitals in Australia, which showed 34.9% and 16.5% of NB using formula during hospitalization, respectively^(22,23)^.

A Brazilian study, conducted in a university hospital, also found that, despite the women and infants being admitted to a rooming-in system, more than 20% of the NB were using artificial milk during the study^(15)^. Considering that rooming-in is recommended when the dyad is healthy and is an important space for breastfeeding guidance and encouragement^(24)^, the data show an exacerbated percentage in the offering of infant formula. The provision of supplements in the maternity ward was associated with a 53% increase in the prevalence of EB interruption in a study in the south of Brazil^(18)^, similarly to this study.

In this sense, Conceição and collaborators sought to identify the reasons for artificial milk indication within a maternity hospital in the State of Rio de Janeiro (Brazil) and found that 64.28% were due to the NB’s conditions (difficulty latching , weak sucking, cleft palate); 10.71% due to maternal conditions; 10.71% without anti-HIV serology; and 14.28% due to other conditions^(15)^. Another study points out that colostrum deficiency (33.8%), difficulty in latching/sucking (23.5%) and “little milk” (70.0%) were the reasons for offering formula to NB within the 48 hours of life in public maternity hospitals in the state of Rio Grande do Norte, Brazil^(10)^.

The following conditions stand out as officially justifiable reasons for early introduction of supplement: prematurity (< 32 weeks), very low weight (< 1,500 g), with risk of hypoglycemia, situations that contraindicate human milk (seropositivity for HIV, HTLV, active herpes in the breast), and adverse clinical conditions in women that pose a barrier to breastfeeding^(25)^. In this study, no characteristics were identified to support the high rate of supplement provision within the maternity ward.

The use of artificial milk in the maternity ward leads many women to keep using it at home, as they do not receive guidance at the time of hospital discharge^(26)^, which contributes to early weaning. When supplement enters the infant’s diet, there is a reduction in breast sucking, which causes a negative impact on maternal milk production, given that lactogenesis III (maintenance of milk production) is autocrine (depends on mechanical stimulus). As the days go by, production can decrease, leading to an increasingly higher supplement offer, and thus the weaning cycle begins^(27)^.

Furthermore, the early use of artificial milk, especially before the NB’s intestinal colonization with the mother’s own milk, can trigger a series of problems for the infant’s health, such as food allergies, gastric discomfort, including diarrhea, increased risk for cardiovascular diseases, obesity, and metabolic diseases^(28)^.

A study shows that the chances of NB to remain exclusively breastfed in the first month, as well as in subsequent months, are increased when they are placed in immediate skin-to-skin contact with the mother and are breastfed within the first hour postpartum (golden hour)^(10)^.

Skin-to-skin contact between the mother and the infant in the first hour of life helps with their adaptation to extrauterine life and cardiorespiratory stability, in addition to favoring contact with the maternal microbiota, for the colonization of the child’s skin as a way of protection. It also stimulates the beginning of breastfeeding, lactogenesis III, and impacts the early creation of a bond between the mother and her child^(29)^. In this study, 61.76% of newborns did not breastfeed in the first hour of life, which may be related to the fact that they were not placed in early skin-to-skin contact.

The factors found to be associated with early weaning are entirely subject to intervention, such as the mother’s belief in using pacifiers to calm the baby. Health professionals play a fundamental role in this process, both in managing difficulties and in solving the many doubts that may arise in the initial lactation process^(14)^.

In this regard, a study showed that prenatal care is the most opportune moment to answer questions and inform pregnant women about breastfeeding, with quality prenatal care being essential, not limited to the basics, also involving the partner and the family in this process^(30)^. To face the factors found in this study, professionals should participate by providing guidance, informing, and promoting security and autonomy to women during prenatal care, rooming-in, and the postpartum period.

Limitations of this study include the fact that analyzed the association between neonatal characteristics and early weaning in health regions of a municipality in the interior of Bahia, Brazil, with its own sociodemographic and cultural characteristics; it is thus not possible to affirm that its findings will replicate in other contexts. Added to this, the fact that the collection was not conducted while the infants were exclusively breastfed may have contributed to memory biases, potentially interfering with study results and acting as a limiting factor.

In short, the findings of this study have potential implications for the construction of subsidies that expand knowledge of infant characteristics interfering with breastfeeding, mainly considering the use of bottles and pacifiers and the introduction of infant formulas within the maternity ward, based on a logistic regression model that managed to explain 28% of early weaning in the sample. This will allow us to think of new strategies to help increase breastfeeding rates. We believe that interventions that help increase maternal knowledge and breastfeeding should be part of breastfeeding promotion strategies.

## CONCLUSION

The use of bottles and pacifiers and the introduction of infant formulas within the maternity ward with continued supplementation after hospital discharge were significantly associated with early weaning in this study’s sample. This fact reveals that timely and prompt identification, in addition to knowledge of these factors, may help recognizing newborns at risk of early weaning and adopting actions to increase the duration of human breastfeeding. Interventions aimed at education are essential to encourage exclusive breastfeeding and should be conducted at different stages of prenatal care, such as the delivery room, rooming-in, and postpartum period.

Therefore, permanent monitoring, associated with continued monitoring, becomes essential in consolidating actions to promote, protect, and support breastfeeding, to reduce infant morbidity and mortality rates. Studies that provide greater depth and involve different populations are expected to expand the scope of this theme to generate solid evidence-based foundations regarding neonatal factors associated with early weaning.
