# Supplementary material for: Neonatal factors associated with early weaning in a municipality in Bahia, Brazil: a cross-sectional study
Source: Rev Esc Enferm USP. 2024 Oct 7;58:e20240091. doi: 10.1590/1980-220X-REEUSP-2024-0091en (PMC11469635; doi:10.1590/1980-220X-REEUSP-2024-0091en)
Supplement: Translation

# Translation: Fatores neonatais associados ao desmame precoce em um município da Bahia: um estudo transversal

**DOI:** 10.1590/1980-220X-REEUSP-2024-0091pt

**Authors:** Aloysia Graça Costa Unfried, Gilvânia Patrícia do Nascimento Paixão, Chalana Duarte de Sena Fraga, Josenilde Damascena Oliveira, Jorge Lopes Cavalcante

**Affiliations:** 1 Universidade do Estado da Bahia, Salvador, BA, Brasil.; 2 Universidade do Estado da Bahia, Senhor do Bonfim, BA, Brasil.; 3 Universidade do Estado da Bahia, Jacobina, BA, Brasil.

**Keywords:** Amamentação, Leite Humano, Lactente, Desmame Precoce, Fórmulas Infantis

## Abstract

**Objetivo:**

Analisar os fatores neonatais associados ao desmame precoce.

**Método:**

Trata-se de um estudo transversal, realizado entre os meses de março e setembro de 2023, com 180 mulheres entre seis meses e dois anos de pós-parto, de um município da Bahia. Para a análise bivariada, foram utilizados os testes do qui-quadrado de Pearson, considerando p < 0,05. A análise ajustada incluiu as variáveis com valor de p < 0,20, mantendo aquelas com p < 0,05, utilizando-se a técnica de *Stepwise* para regressão logística múltipla, com intervalo de confiança de 95%.

**Resultados:**

A oferta de bicos artificiais chupetas/mamadeiras (OR: 18,96; IC 95%: 7,68–46,79; p < 0,001) e oferta de complemento ainda na maternidade (OR: 4,44; IC95%: 1,76–11,17; p: 0,002) estiveram associados a maiores chances de desmame precoce.

**Conclusão:**

Hábitos e crenças, como o uso de mamadeira e chupetas, e a introdução de fórmulas infantis ainda dentro da maternidade com continuidade da suplementação após a alta hospitalar foram os fatores neonatais associados ao desmame precoce no contexto estudado.

## INTRODUÇÃO

Fonte de nutrientes para o crescimento e desenvolvimento da criança, o leite humano é composto por ingredientes diversos e complexos. Dentre esses, estão os macronutrientes, como carboidratos (lactose), gorduras, proteínas, inúmeras vitaminas e sais minerais, assim como fatores bioativos, imunoglobulinas, metabólitos e microrganismos com papéis fisiológicos específicos ao leite humano^(1)^.

Estudos apontam diversas evidências dos seus efeitos ainda na infância, especialmente na redução das taxas de mortalidade^(2)^ causadas por infecções do trato respiratório e gastrointestinais^(3)^. Na fase adulta, tem-se os efeitos positivos no desenvolvimento e integridade da cavidade oral, reduzindo as taxas de má oclusão dentária, além de benefícios outros, como menor chance de comorbidades crônicas, a exemplo do sobrepeso e diabetes^(4,5)^.

Nesse sentido, a Organização Mundial da Saúde (OMS) e o Ministério da Saúde (MS) recomendam que o Aleitamento Exclusivo (AE) seja mantido até o sexto mês de vida, diretamente da mama ou ordenhado, sem outros sólidos, e com introdução da alimentação complementar a partir desse período, com manutenção do aleitamento por período igual ou superior a dois anos^(6)^.

O Brasil é referência mundial para o aleitamento humano (AH), em função das políticas públicas adotadas há pelo menos 30 anos no tocante ao estímulo à amamentação^(7)^. Conforme o Estudo Nacional de Alimentação e Nutrição (ENANI) realizado entre fevereiro de 2019 e março de 2020, nesses últimos 30 anos houve um aumento importante na prevalência do AE até os seis meses, passando de 2,9% para 45,7%. Nesse mesmo tempo, ocorreu também redução da incidência de doenças diarreicas e infecções respiratórias, ao passo que a expectativa de vida das crianças brasileiras aumentou^(8)^.

No entanto, mesmo com a melhora nos índices da prevalência de AE no Brasil, as taxas ainda não atendem às recomendações atuais da OMS, de 55% de lactentes em AE até o ano de 2025 e 70% para 2030, com a prática mais reduzida sobretudo nas regiões Nordeste (39,0%) e Norte (40,3%)^(6,7,8)^.

Diante dos impactos positivos produzidos pelo AE até os seis meses de vida do neonato, bem como das variações nas taxas de prevalência nas regiões brasileiras, faz-se necessária a realização de estudos mais regionalizados, com intuito de identificar e entender os fatores preditores ao desmame precoce, de forma individualizada, diante dos inúmeros fatores neonatais que podem interferir no manejo do AE. Apesar da heterogeneidade dos estudos e das especificidades presentes nas distintas regiões do Brasil, estudos recentes^(9,10)^ apontam o uso de mamadeiras, chupetas e a introdução precoce de fórmulas como potenciais preditores neonatais do desmame precoce.

A clareza sobre tais características poderá direcionar a adequação das linhas de cuidado e acompanhamento por parte dos profissionais de saúde no período do pré-natal, pós-parto e puericultura, a fim de corroborar a comunidade científica acerca da prevenção de fatores modificáveis que podem ter como consequência a suspensão precoce do aleitamento humano. Dessa forma, delineou-se a seguinte questão de pesquisa: quais são os fatores neonatais associados ao desmame precoce? O objetivo foi analisar os fatores neonatais associados ao desmame precoce.

## MÉTODO

### Desenho do Estudo

Trata-se de um estudo transversal de abordagem quantitativa. Seguiram-se as recomendações do guia *Strengthening the reporting of observational studies in epidemiology* (STROBE).

### Local, População e Critérios de Seleção

O estudo foi realizado em Senhor do Bonfim – BA, município situado no centro Norte Baiano, que apresenta uma área territorial de 789,361 km, 79.813 habitantes, densidade demográfica de 94,413 habitantes/km^2^ e 17,69 óbitos por mil nascidos vivos^(11)^.

A população do estudo foi constituída por 180 binômios vinculados às Estratégia de Saúde da Família (ESF) do município. Foram incluídas mulheres que estivessem entre seis meses e um dia, até dois anos (24 meses) do pós-parto, com filho vivo, contabilizado a partir da data da aplicação do formulário, maiores de 18 anos e cadastradas em ESF do referido município. Não foram incluídas mulheres que possuíam transtornos mentais graves ou que por recomendação médica não puderam amamentar, critérios referidos pelos profissionais da ESF. Com base nos critérios, a amostra foi selecionada pela ESF, que indicava as pessoas que se enquadravam no estudo.

### Cálculo do Tamanho da Amostra

A amostra deste estudo resultou do cálculo amostral realizado através de uma amostra aleatória simples sem reposição para populações finitas. Foi utilizada a calculadora *Survey Monkey* (https:pt.surveymonkey.com/mp/sample-size-calculator/). Considerou-se um tamanho populacional de 951 nascidos vivos, com base no relatório de consolidação da ficha de Agentes Comunitários de Saúde (ACS) do município de Senhor do Bonfim no ano de 2022, poder de 80% e um alfa de 5%, o que contabilizou um total de 140 indivíduos. Contudo, considerando a quantidade de variáveis independentes a serem investigadas, foram adicionados mais 40 indivíduos, o que perfez um total de 180 participantes. De forma proporcional ao número de residentes na zona urbana e rural do município, foram investigadas 69 e 111 mulheres, respectivamente.

### Coleta de Dados

Para a coleta de dados, primeiramente foi realizado um teste piloto, com a finalidade de testar e adequar o instrumento que foi desenvolvido pelos pesquisadores, tomando como base as publicações mais recentes na área materno-infantil e orientações do MS e OMS. O teste foi aplicado a 10 mulheres incluídas nos critérios de elegibilidade e readaptado diante das dificuldades percebidas durante a aplicação, com posterior descarte.

Inicialmente houve conversa prévia com a enfermeira de cada ESF, com levantamento das mulheres que se enquadravam na população alvo do estudo. Posteriormente, foi disponibilizado contato da equipe de ACS. Por meio de contato telefônico, e após confirmação do interesse em participar da pesquisa, os agentes programavam dia e horário para realização de visita ao domicílio de cada mulher elegível para o estudo.

A coleta de dados ocorreu individualmente entre os meses de março a setembro de 2023 por três pesquisadoras devidamente treinadas em um único encontro no domicílio da participante. Após a apresentação do objetivo da pesquisa e assinatura em duas vias do Termo de Consentimento Livre e Esclarecido (TCLE), ocorreu a aplicação do instrumento de pesquisa, ainda não validado. O questionário aplicado possui 63 perguntas fechadas (múltipla escolha ou dicotômicas), com informações sobre as características sociodemográficas, obstétricas, hábito de vida, informações sobre o lactente e assistência profissional. A seleção das variáveis para este manuscrito foi realizada a partir do seu objetivo, ou seja, foram selecionadas as variáveis do lactente (dados neonatais), perfazendo um total de 14 variáveis que são descritas a seguir.

A variável desfecho analisada no estudo foi desmame precoce (suspensão do aleitamento em período inferior a seis meses), estruturado de acordo com a pergunta: Suspendeu o aleitamento humano exclusivo antes dos seis meses (não, sim).

As Variáveis relacionadas ao lactente foram: Sexo (feminino, masculino), idade atual (6 a 12 meses,13 a 24 meses), peso ao nascimento (menor que 2,5 kg, entre 2,5 kg e 3 kg, entre 3 e 4,85 kg), idade gestacional no parto entre 37 e 41 semanas (não, sim), contato pele a pele na primeira hora (não, sim), tempo que iniciou o aleitamento pós-parto (até 1 hora, após 1 hora), recebeu outro tipo de leite na maternidade (não, sim), aleitamento em livre demanda (não, sim), bebê tinha dificuldade na sucção (não, sim), recebeu outro leite por indicação médica (não, sim), utilizou chupeta ou mamadeira antes dos 6 meses (não, sim), introduziu chupeta ou mamadeira na maternidade (não, sim), bebê já precisou ficar hospitalizado (não, sim), realizou até 6 consultas de puericultura (não, sim).

### Análise e Tratamento dos Dados

Os dados foram digitados no programa Microsoft Excel® 2010 com posterior exportação para a análise no programa estatístico STATA, versão 15.0 (*Stata Corp.,* USA). As variáveis independentes com p < 0,05 na análise bivariada, utilizando-se o teste do qui-quadrado de Pearson, foram selecionadas e incluídas na análise multivariada. Nas análises multivariadas, consideraram-se conjuntamente as variáveis independentes cuja associação na análise bruta foi significante ao nível de 20% (p ≤ 0,20).

Como método de seleção de variáveis para o modelo final, foi utilizada a técnica de *Stepwise* para regressão logística múltipla. Dessa forma a análise ajustada incluiu as variáveis independentes com valor de p < 0,20, sendo mantidas no modelo final as variáveis que apresentaram associação estatisticamente significativa com o desfecho ‘desmame precoce’ em um nível de 5% (p < 0,05).

Obtiveram-se assim as estimativas brutas de razões de chance (*odds ratio*-OR) referentes à associação entre a variável dependente (desmame precoce) e independentes (fatores neonatais), assim como as razões de chance ajustadas no modelo final e seus respectivos intervalos de confiança de 95% (IC 95%).

### Aspectos Éticos

O estudo possui vinculação ao Projeto de Pesquisa e Extensão: Grupo de Apoio ao Aleitamento Materno (GAAM), desenvolvido na Universidade do Estado da Bahia, Campus VII, e foi aprovado pelo Comitê de Ética em Pesquisas da Universidade do Estado da Bahia, no ano de 2022, sob o parecer de número 5.437.925 e CAAE 84122117.8.0000.0057. Foram utilizados o Termo de Consentimento Livre e Esclarecido (TCLE) para todas as participantes e respeitados os princípios éticos de pesquisa fundamentados na Resolução N° 466/12 do Conselho Nacional de Saúde.

## Resultados

Fizeram parte da pesquisa 180 mulheres. A maioria das participantes tinha idade entre 16 e 29 anos, com nível escolar médio, eram casadas ou viviam em união estável, se autodeclararam pardas ou pretas, exerciam atividades do lar e referiam receber auxílio de programas sociais. Residiam em zona urbana com esposo e filhos, sem necessidade de transporte para atendimento na UBS, e tiveram acesso à informação sobre aleitamento através das consultas de pré-natal. Vale destacar que uma parcela de 13,89% das entrevistadas afirmou fazer uso de álcool ou cigarro durante a gestação/pós-parto. A prevalência do desmame precoce na amostra estudada foi de 48,33% (n = 87).

A Tabela 1 apresenta a análise bruta da associação entre as características neonatais e o desmame precoce. Observou-se que os seguintes fatores se associaram positivamente ao desfecho: utilizou chupeta e/ou mamadeira (OR: 16,36; IC 95%: 7,06–37,88; p < 0,001); introduziu chupeta e/ou mamadeira na maternidade (OR: 3,58; IC 95%: 0,13–0,57; p: 0,001); recebeu outro leite por indicação médica (OR: 14,72; IC 95%: 1,8–115,8; p: 0,001); recebeu outro tipo de leite na maternidade (OR: 3,28; IC 95%: 1,60–6,71; p: 0,001), tempo de início do aleitamento no pós-parto (OR: 0,41; IC 95%: 1,29–4,46; p: 0,005); aleitamento realizado em livre demanda (OR: 0,14; IC 95%: 0,01–1,24; p: 0,044).

**Tabela 1:** Análise bruta dos aspectos relacionados ao lactente, segundo desmame precoce* – Senhor do Bonfim, BA,Brasil, 2023 (n = 180).

Características neonatais	Desmame precoce				P	OR bruto (IC 95%)	
	Não		Sim				
	n	%	N	%			
**Sexo da criança**					0,762		
Feminino	47	52,81	42	47,19		1	
Masculino	46	50,55	45	49,45		1,09(0,60–1,96)	
**Idade atual da criança**					0,821		
Seis meses a um ano	38	50,67	37	49,33		1	
Treze a vinte e quatro meses	55	52,38	50	47,62		0,93(0,51–1,68)	
**Peso no nascimento**					0,406		
Abaixo de 2.500 g	09	50,00	09	50,00		1	
Entre 2.500 g e 3.000 g	23	44,23	29	55,77		1,26(0,43–3,69)	
Entre 3.000 g e 4.850 g	61	55,45	49	44,55			
**Idade gestacional no parto foi entre 37 e 41 semanas**					0,492		
Não	06	42,86	08	57,14		1	
Sim	87	52,41	79	47,59		0,68(0,22–2,04)	
**Contato pele a pele na primeira hora**					0,077***		
Não	21	41,18	30	58,82		1	
Sim	72	55,81	57	44,19		0,55(0,28–1,06)	
**Tempo que iniciou o aleitamento no pós-parto**					0,005**		
Até 1 hora pós-parto	67	59,82	45	40,18		1	
Após 1 hora pós-parto	26	38,24	42	61,76		2,40(1,29–4,46)	
**Recebeu outro leite na maternidade**					0,001**		
Não	79	58,96	55	41,04		1	
Sim	14	30,43	32	69,57		3,28(1,60–6,71)	
**Aleitamento realizado em livre demanda**					0,044***		
Não	01	14,29	06	85,71		1	
Sim	92	53,18	81	46,82		0,14(0,01–1,24)	
**Bebê apresentou problemas de sucção**					0,218		
Não	82	53,59	71	46,41		1	
Sim	11	40,74	16	59,26		1,67(0,73–3,85)	
**Recebeu outro leite por indicação médica**					0,001**		
Não	92	55,09	75	44,91		1	
Sim	01	07,69	12	92,31		14,72(1,8–115,8)	
**Bebê já ficou hospitalizado**					0,321		
Não	79	49,69	80	50,31		1	
Sim	08	38,10	13	61,90		0,62(0,24–1,58)	
**Utilizou chupeta e/ou mamadeira**					<0,001**		
Não	58	87,88	08	12,12		1	
Sim	35	30,70	79	69,30		16,36(7,06–37,88)	
**Introduziu chupeta e/ou mamadeira na maternidade**					0,001**		
Não	80	59,26	55	40,74		1	
Sim	13	28,89	32	71,11		3,58(0,72–7,43)	
**Realizou até seis consultas de puericultura**					0,496		
Não	52	49,52	53	50,48		1	
Sim	41	54,67	34	45,33		0,81(0,44–1,47)	

Nota: *Suspensão do aleitamento exclusivo antes dos seis meses de idade

**p < 0,05.

***p < 0,20.

Na Tabela 2, é apresentada a análise de regressão logística múltipla ajustada dos aspectos neonatais associados ao desfecho de desmame precoce. Encontrou-se que o lactente ao qual foi oferecido chupeta e/ou mamadeira, e aqueles que receberam outro tipo de leite ainda na maternidade, apresentaram 18,96 (IC 95%: 7,68–46,79; p < 0,001) e 4,44 (IC 95%: 1,76–11,17; p: 0,022) vezes mais chances de apresentarem desmame precoce. Cabe destacar que este modelo final de regressão logística foi capaz de explicar em 28% (chi^2^ = 0,2868) a associação das variáveis independentes (Utilizou chupeta e/ou mamadeira/ Recebeu outro leite na maternidade) com a variável dependente (desmame precoce).

**Tabela 2:** Análise de regressão logística ajustada dos fatores neonatais, associados ao desfecho de desmame precoce* – Senhor do Bonfim, BA, Brasil, 2023 (n = 180).

Variáveis	N (%)	OR ajustado (IC 95%)	P
**Utilizou chupeta e/ou mamadeira**			<0,001
Não	66(36,67)	1	
Sim	114(63,33)	18,96(7,68–46,79)	
**Recebeu outro leite na maternidade**			0,002
Não	134(74,44)	1	
Sim	46(25,56)	4,44(1,76–11,17)	

Nota: *Suspensão do aleitamento exclusivo antes dos seis meses de idade.

## DISCUSSÃO

O objetivo do presente estudo foi analisar os fatores neonatais associados ao desmame precoce em uma amostra de 180 mulheres do interior do Estado da Bahia, Nordeste do Brasil. Dentre os fatores neonatais analisados neste estudo, a utilização de chupeta e/ou mamadeira e ter recebido outro tipo de leite ainda na maternidade se associaram positivamente ao desmame precoce.

Chama atenção que 48,33% dos lactentes foram desmamados precocemente, porcentagem inferior às estimadas em geral no contexto brasileiro, que correspondem a 54,3%^(8)^. Esse importante achado revelou que mais da metade dos lactentes recebeu aleitamento exclusivo até os 6 meses de vida (51,67%). Isso demostra que, na região avaliada nesta pesquisa, o índice de aleitamento exclusivo foi superior ao encontrado no país, entre os anos 1986 e 2020^(8)^.

Das variáveis que se associaram positivamente ao desfecho do estudo, a utilização de chupeta e/ou mamadeira se apresentou mais fortemente associada ao desmame precoce. Dessa forma, as mulheres que relataram o oferecimento desses acessórios tiveram 18,96 vezes a chances em desmamar precocemente seu filho quando comparadas àquelas que não ofereceram bicos artificiais. A prevalência do uso de bicos artificiais entre lactentes menores de 24 meses é de 52,1% no Brasil e de 55,8% na região Nordeste^(8)^. Em nosso estudo, o uso de chupetas e/ou mamadeiras teve uma prevalência de 63,34%.

A descontinuidade do aleitamento exclusivo pode ser devida a fatores socioeconômicos e de saúde, além dos fatores biológicos^(10)^. A literatura vem apontando que a falta de incentivo por parte das instituições de saúde, falta de apoio familiar às nutrizes e a oferta de bicos (chupeta e/ou mamadeiras) estão entre as principais causas do desmame precoce^(12,13)^.

As razões para a introdução de chupeta precisam ser definidas. Ao longo das gerações, a sua oferta foi atrelada a um fator cultural: seu uso tinha como finalidade acalmar o lactente em momentos de choro. Contudo, além da confusão de bicos, a utilização do artifício da chupeta como tranquilizador do lactente pode interferir diretamente na produção e na quantidade do leite humano, pois está diretamente relacionada à redução das mamadas, ou seja, quanto maior a frequência do aleitamento, mais produção de leite^(14,15)^.

O cenário brasileiro não difere do internacional em relação à influência negativa do uso de chupetas. Pesquisa realizada em Roma com 542 mulheres identificou que lactentes que utilizaram chupetas nas primeiras 2 semanas de vida tiveram 2,39 vezes mais risco de interromper o aleitamento quando comparadas às que não as utilizaram^(16)^.

Adicionado a complicação do desmame precoce, o uso de bicos artificiais promove alterações no padrão respiratório, favorecendo a respiração bucal, levando ao comprometimento da fala e a ocorrência de má oclusão, como mordida aberta ou cruzada. Entre outros aspectos negativos para a saúde do lactente, estão episódios de otite média aguda, alterações de palato, maxilar e comprometimento imunológico, por serem considerados potenciais reservatórios de contaminação, ocasionando febre, diarreias, aftas e candidíase^(15,17)^.

Tais resultados evidenciam a relevância do desencorajamento do uso da chupeta dentro da atuação profissional, visando a manutenção do AE pelo tempo recomendado^(12)^. Ademais, a prática do uso de bicos artificiais é desaconselhável, pois representa meio de contaminação, interfere no aleitamento por livre demanda, reduz o tempo de sucção das mamas e afeta o desenvolvimento natural da deglutição, além de retardar o tempo do estabelecimento da lactação^(6,18)^.

Outra variável fortemente associada ao desfecho e preditora do desmame precoce na população estudada foi ter recebido outro tipo de leite na maternidade. Esses lactentes apresentaram 4,44 vezes a chance de serem desmamados precocemente quando comparados àqueles que foram alimentados com leite humano nesse período. Neste estudo, foi encontrada a prevalência de 25,56% de lactentes que receberam complemento nas primeiras horas pós-parto.

O complemento é caracterizado pela oferta de leite humano pasteurizado ou fórmula infantil, na ausência do primeiro, para recém-nascido (RN) em condições diversas^(19)^. Apesar dos esforços na manutenção do aleitamento exclusivo, a oferta da alimentação complementar, com predomínio de fórmulas infantis, ainda é uma prática frequente e sem razões justificáveis pela Iniciativa Hospital Amigo da Criança (IHAC), dentro das primeiras horas de um RN de baixo risco^(20)^.

Instituições Nacionais e Internacionais fazem uso rotineiro de complemento no pós-parto^(15)^, o que é evidenciado em diversos estudos, a saber: maternidades públicas da cidade de Natal, Rio Grande do Norte apresentaram frequência de 57,6% no uso de suplemento na primeira hora de vida^(19)^; maternidade do Rio de Janeiro, com prevalência de 12% dos RNs que receberam complemento lácteo durante internação^(21)^, além de maternidades no Irã e cinco hospitais na Austrália, que apresentaram 34,9% e 16,5% de RNs em uso de fórmula durante o internamento, respectivamente^(22,23)^.

Estudo Nacional, realizado em um Hospital Universitário, constatou ainda que, apesar de as mulheres e lactentes estarem internados em sistema de alojamento conjunto, mais de 20% dos RNs estavam em uso de leite artificial durante a realização da pesquisa^(15)^. Levando-se em consideração que o alojamento conjunto é recomendado quando o binômio é sadio, sendo importante espaço de orientação e incentivo ao aleitamento^(24)^, os dados mostram um percentual exacerbado no oferecimento de fórmula infantil. A oferta de complemento na maternidade foi associada a um aumento de 53% na prevalência de interrupção do AE, em estudo no Sul do Brasil^(18)^, assim como o que ocorreu nesta pesquisa.

Nesse sentido, Conceição e colaboradores buscaram identificar os motivos das indicações para o uso do leite artificial dentro de uma maternidade no Estado do Rio de janeiro (Brasil) e constataram que se deram em: 64,28% por condições do RN (dificuldade de pega, sucção débil, fenda palatina); 10,71% por condições maternas; 10,71% sem sorologia anti-HIV; e 14,28% por outras condições^(15)^. Outro estudo aponta que deficiência de colostro (33,8%), dificuldade de pega/sucção (23,5%) e “pouco leite” (70,0%), foram os motivos identificados para o oferecimento de fórmula ao RN dentro das 48 horas de vida em maternidades públicas no estado do Rio Grande do Norte^(10)^.

Ressaltam-se como razões oficialmente justificáveis para introdução precoce de complemento as seguintes condições: prematuridade (< 32 semanas), muito baixo peso (< 1.500 g), com risco para hipoglicemia, situações que contraindiquem o leite humano (soropositividade para HIV, HTLV, herpes ativa na mama) e condições clínicas adversas da mulher que seja barreira ao aleitamento^(25)^. Neste estudo não foram identificadas características que abonem o alto índice na oferta de complemento ainda dentro da maternidade.

É importante salientar que o uso de leite artificial na maternidade faz com que muitas mulheres mantenham a oferta no domicílio, uma vez que não recebem orientação no momento da alta hospitalar^(26)^, o que contribui para o desmame precoce. Quando um complemento entra na alimentação do lactente, há redução da sucção da mama pelo RN, o que causa impacto negativo na produção láctea materna, haja vista que a lactogênese III (manutenção da produção láctea) é autócrina (depende do estímulo mecânico). Desse modo, com o passar dos dias, a produção pode efetivamente diminuir, o que faz com que cada vez se ofereça mais complemento e assim, inicia-se o ciclo de desmame^(27)^.

Além disso, o uso precoce de leite artificial, principalmente antes da colonização intestinal do RN pelo leite da própria mãe, pode desencadear uma série de problemas para a saúde do lactente, como alergias alimentares, desconfortos gástricos, incluindo diarreias, aumento do risco para doenças cardiovasculares, obesidade e doenças metabólicas^(28)^.

Estudo demonstra que as chances de permanecer em aleitamento exclusivo no primeiro mês, bem como nos meses subsequentes, são aumentadas quando o RN é colocado em contato imediato com a genitora, pele a pele, melhorando os índices quando é possível o aleitamento acontecer no seio dentro da primeira hora pós-parto (*golden hour*)^(10)^.

O contato pele-a-pele entre a mãe e o lactente na primeira hora de vida ajuda na sua adaptação à vida extrauterina e na estabilidade cardiorrespiratória, além de favorecer o contato com a microbiota materna, para a colonização da pele do filho como forma de proteção. Também estimula o início da amamentação, a lactogênese III e repercute na criação precoce de vínculo entre a mãe e seu filho^(29)^. Nesta pesquisa, 61,76% dos RN não amamentaram na primeira hora de vida, o que pode se relacionar ao fato de não terem sido colocados em contato precoce pele a pele.

Os fatores encontrados que se associaram ao desmame precoce são totalmente passíveis de intervenção, a exemplo da crença materna do uso de chupetas para acalmar o bebê.. Profissionais de saúde têm papel fundamental nesse processo, tanto no manejo de dificuldades quanto para sanar as muitas dúvidas que podem surgir no processo inicial de lactação^(14)^.

A esse respeito, estudo destacou que o pré-natal é o momento mais oportuno para esclarecer as dúvidas e informar às gestantes sobre aleitamento, sendo indispensável a execução de um pré-natal de qualidade, não limitado ao básico, envolvendo também neste processo o parceiro e a família^(30)^. Nesse sentido, para enfrentar os fatores encontrados nesse estudo, é importante a participação e orientação profissional, fornecendo informação, segurança e autonomia às mulheres, durante o pré-natal, em alojamento conjunto e puerpério.

Como limitadores desta pesquisa, cita-se o fato de tratar-se de um estudo que analisou a associação entre as características neonatais e o desmame precoce em regiões de saúde de um município do interior da Bahia, com características sociodemográficas e culturais próprias, não sendo possível afirmar que os fatores aqui encontrados possam se repetir em outros contextos. Adicionado a isso, o fato de a coleta não ter sido realizada enquanto os lactentes estavam em aleitamento exclusivo pode ter contribuído para os vieses de memória, potenciais interferentes no resultado da pesquisa, se comportando como um limitador.

Em suma, os achados do presente estudo têm implicações potenciais na construção de subsídios que ampliam o conhecimento a respeito dos fatores relacionados ao lactente interferentes no aleitamento humano, principalmente considerando o uso de mamadeira e chupetas, e a introdução de fórmulas infantis ainda dentro da maternidade, com base num modelo de regressão logística que conseguiu explicar em 28% o desmame precoce na amostra estudada. Isso permitirá pensar em novas estratégias para auxiliar o incremento dos índices de amamentação. Acreditamos que intervenções que ajudem a elevar o conhecimento materno e a amamentação devam fazer parte das estratégias de promoção do aleitamento humano.

## CONCLUSÃO

O uso de mamadeira e chupetas e a introdução de fórmulas infantis ainda dentro da maternidade com continuidade da suplementação após alta hospitalar foram significativamente associados ao desmame precoce na amostra estudada. Esse fato revela que a identificação oportuna e em tempo hábil, além dos conhecimentos desses fatores, podem reconhecer os neonatos que esLtão em risco de desmame precoce e ajudar a construir ações capazes de aumentar a duração do aleitamento humano. Intervenções direcionadas à educação são essenciais para o incentivo ao aleitamento exclusivo, devendo ser realizada em diferentes etapas do pré-natal, como sala de parto, no alojamento conjunto e puerpério.

Assim, o acompanhamento permanente, associado à vigilância continuada, torna-se imprescindível na consolidação das ações de promoção, da proteção e do apoio ao aleitamento humano, a fim de reduzir as taxas de morbimortalidade infantil. Espera-se ampliar o escopo dessa temática com estudos que produzam maior aprofundamento e com uma população ampliada a diferentes realidades, a fim de gerar uma base de evidências sólidas a respeito dos fatores neonatais associados ao desmame precoce.
